# ATMLP Enhances Radioresistance in Non-Small Cell Lung Cancer through AKT-Mediated Lipid Droplet Accumulation

**DOI:** 10.7150/ijbs.116401

**Published:** 2026-01-21

**Authors:** Yingchu Dai, Wanyi Wu, Tingyu Jiao, Lu Hou, Wanshi Li, Jiyuan Liu, Qianjiale Gu, Fengtao Su, Jing Nie, Bingyan Li, Jing Wang, Hailong Pei, Guangming Zhou

**Affiliations:** 1 Department of Radiotherapy and Oncology, Affiliated Hospital of Jiangnan University, Wuxi 214122, China.; 2 State Key Laboratory of Radiation Medicine and Protection, School of Radiation Medicine and Protection, Medical College of Soochow University, Suzhou 215123, China.; 3 Collaborative Innovation Center of Radiological Medicine of Jiangsu Higher Education Institutions, Suzhou 215123, China.; 4 Department of Nuclear Medicine, Affiliated Hospital of Jiangnan University, No. 1000, Hefeng Road, Wuxi, 214000, China.; 5 National Key Laboratory for Metrology and Calibration Techniques, China Institute of Atomic Energy, P. O. Box 275-20, Beijing 102413, China.; 6 Cancer Institute, Fudan University Shanghai Cancer Center, Shanghai 200032, China.; 7 General Surgery Department, Beijing Shijitan Hospital, Capital Medical University, Beijing, China.

**Keywords:** ionizing radiation, AKT signaling pathway, lipid droplet, lipid metabolism, radioresistance

## Abstract

Radioresistance remains a critical barrier to successful radiotherapy in non-small cell lung cancer (NSCLC). ATMLP, a mitochondrial-localized peptide encoded by lncRNA AFAP1-AS1, has been previously associated with tumor progression. In this study, we uncover a previously unrecognized role of ATMLP in promoting radioresistance by facilitating intracellular lipid droplet (LD) accumulation through AKT pathway activation. Mechanistically, ATMLP reduces radiation-induced reactive oxygen species (ROS) accumulation, thereby relieving ROS-mediated suppression of AKT phosphorylation, which in turn enhances lipid storage and promotes tumor cell survival under ionizing radiation. Genetic knockout of ATMLP leads to excessive ROS generation, impaired AKT activation, and diminished LD accumulation, ultimately sensitizing NSCLC cells to radiation. Conversely, ATMLP overexpression decreases ROS levels, increases post-radiation clonogenicity, and accelerates tumor growth. Inhibition of the AKT pathway abrogates ATMLP-induced lipid accumulation and reverses the radioresistant phenotype. These findings identify ATMLP as a key mediator linking ROS homeostasis and lipid metabolic reprogramming to radiation response, and suggest that targeting the ATMLP-AKT axis may represent a promising therapeutic strategy to enhance radiotherapy efficacy in NSCLC.

## Introduction

Lung cancer, the leading cause of cancer-related mortality worldwide, presents a formidable challenge in oncology, with non-small cell lung cancer (NSCLC) representing the majority of cases 1. Despite the pivotal role of radiation therapy in NSCLC management, therapeutic outcomes are frequently compromised by the intrinsic radioresistance of tumor cells 2. This resistance significantly affects the prognosis of lung cancer patients, underscoring the critical need for a deeper understanding of the underlying mechanisms of NSCLC pathogenesis, progression, and resistance.

Metabolic reprogramming has emerged as a hallmark of cancer, with lipid metabolism playing a pivotal role in tumor survival and therapy resistance 3,4. Lipid droplet (LD), previously viewed as static storage organelles, are now recognized as dynamic hubs influencing energy balance, redox homeostasis, and signal transduction. These functions are critical for cancer cell survival, particularly under the oxidative and metabolic stress induced by radiation therapy 5,6.

The role of long non-coding RNAs (lncRNAs) in cancer has evolved from being perceived as transcriptional noise to crucial regulatory molecules of cancer biology, including metabolic reprogramming 7. Notably, some lncRNAs encode functional peptides that directly impact cellular metabolism. For instance, MOXI, a peptide encoded by lncRNA LINC00116, modulates mitochondrial fatty acid β-oxidation 8. Similarly, our prior work identified a mitochondrial-localized peptide, ATMLP (lncRNA *AFAP1-AS1* translated mitochondrial-localized peptide), encoded by the lncRNA AFAP1-AS1 9. While ATMLP has been implicated in promoting autophagic dysfunction, its role in lipid metabolism and radioresistance remains unexplored.

This study suggests that ATMLP may play a significant role in modulating NSCLC's metabolic adaptation and consequent radioresistance. Specifically, ATMLP appears to enhance lipid droplet accumulation through the activation of the AKT signaling pathway, facilitating tumor cell survival under radiative stress. By delving into the molecular interactions between ATMLP expression, lipid metabolism, and radioresistance, we aim to uncover potential therapeutic targets that could disrupt these adaptive survival mechanisms in NSCLC to enhance radiotherapy efficacy.

## Materials and Methods

### Cell lines

We utilized several human cell lines for our experiments: NSCLC lines A549, H1299, Calu-1; human small airway epithelial cells (HSAEC1-KT); cervical cancer cells (HeLa); normal human liver cells (L02); and human embryonic kidney cells (HEK293T). These cell lines were procured from the Typical Cultures Depository Center, Rockville, MD, USA, and were cultured according to the American Type Culture Collection (ATCC) guidelines. Each line was used within 10-20 generations to ensure viability and consistency. Routine testing for mycoplasma contamination was conducted using a detection kit (Beyotime, Guangzhou, China). Cells were cultured in DMEM or RPMI-1640 supplemented with 10% fetal bovine serum and maintained at 37°C in a 5% CO2 atmosphere. Irradiation was performed using a RAD SOURCE RS 2000 X-ray machine (maximum tube voltage 160 kV, maximum current 25 mA) at a dose rate of 1.225 Gy/min under room temperature conditions, with total doses of 2, 4, 6, and 8 Gy.

### Clinical specimens

NSCLC tissues and matched adjacent normal tissues were sourced from the Second Affiliated Hospital of Soochow University. These samples were immunostained using an ATMLP antibody (Abclone, Wuhan, China). The use of these human tissues received ethical clearance from the Ethics Committee of Soochow University (Approval No. SUDA20211117A01), with written informed consent from all donors or their next of kin.

### Animal experimentation

The xenograft model involved subcutaneous injection of 1×10^6^ A549 cells into the flanks of 8-week-old nude mice (n = 5). After the tumors grew to the appropriate size, they were treated with AKT inhibitors, ionizing radiation therapy treatments, respectively, according to the experimental procedure, and the tumor growth was recorded. Then, mice were euthanized; tumors were excised, fixed in 4% formalin, processed for histology, and stained with oil red. All animal procedures were approved by the Institutional Review Board and Animal Care and Use Committee of Soochow University and conducted in the SPF Animal Laboratory at Soochow University, adhering to ethical guidelines for animal welfare. Irradiation was conducted using a PXi X-RAD 225Cx/SmART small animal irradiator (maximum tube voltage 225 kV, open-field dose rate 4.48 Gy/min) with a total dose of 4 Gy.

### Gene silencing and overexpression

To investigate the role of ATMLP in non-small cell lung cancer (NSCLC), we utilized CRISPR/Cas9 technology to generate ATMLP knockout (KO) cell lines. Guide RNAs (gRNAs) targeting the ATMLP coding region were designed using an online CRISPR design tool. These gRNAs were cloned into a vector expressing Cas9 nuclease. Successful knockouts were confirmed via sequencing and Western blot to ensure the absence of ATMLP protein expression. To knock out ATMLP in cells, the following gRNA pair flanking ATMLP: gRNA-1: GCGGCTATTGAAGTGAACGCCGG; gRNA-2: TCACTTCAATAGCCGCTCGAAGG; gRNA-3: AAAGGACCTATTGCTCACCA; gRNA-4: AACGCCGGTATGAAGGGTGT was used. For overexpression studies, cells were transfected with pcDNA 3.1-ATMLP, EGFP-C1/EGFP-N1-ATMLP, or pLV-U6-PolyA plasmids using Lipofectamine 3000. Additionally, to overexpress ATMLP, we also constructed lentiviral vectors containing the full-length coding sequence of ATMLP under the control of a CMV promoter. NSCLC cells were transduced with these lentiviral vectors, and stable overexpressing cell lines were selected using puromycin. Overexpression was verified by Western blot analysis, confirming the increased levels of ATMLP mRNA and protein. These genetically modified cell lines provided the basis for subsequent experiments assessing the impact of ATMLP expression on the radioresistance of NSCLC cells. To minimize off-target effects, multiple gRNAs were designed targeting ATMLP, and two independent knockout clones were established. Loss of ATMLP expression was validated by Western blotting. All functional assays were performed in both clones, and results were consistent across lines.

### Western blotting

Cells were lysed using RIPA lysis buffer (Beyotime, Hangzhou) supplemented with protease inhibitors (Beyotime, Hangzhou). Lysates were boiled and then centrifuged at 13,000 rpm for 10 minutes to remove debris. Protein concentrations were determined using the BCA Protein Assay Kit (Beyotime, Hangzhou). Equal amounts of protein were separated by SDS-PAGE and transferred onto PVDF membranes. The membranes were blocked with 5% non-fat milk and incubated with primary antibodies overnight at 4°C. The primary antibodies used included anti-GAPDH (1:3000, Proteintech), anti-ATMLP (1:500, Abclonal), anti-β-actin (1:1000, CST), anti-PI3K (1:1000, CST), anti-AKT (1:1000, CST), and anti-phospho-AKT (Ser473) (1:1000, CST). Membranes were washed and incubated with HRP-conjugated secondary antibodies. Protein bands were visualized using enhanced chemiluminescence and imaged using a chemiluminescence detection system. Band intensities were quantified with image analysis software to assess the relative expression levels and activation status of the proteins.

### Oil red staining

For lipid visualization, cells or tissue sections were fixed and stained with Oil Red O solution (0.5% in isopropanol) diluted with water (3:2 v/v). After staining for 15 minutes, samples were washed with 60% isopropanol and counterstained with hematoxylin if needed. Lipids appeared red, and nuclei appeared blue under a light microscope. Lipid droplet accumulation was quantified using ImageJ software. For each condition, Oil Red O-stained images were analyzed, and grayscale values corresponding to both droplet size and intensity were measured. At least 100 cells per group were analyzed, and results were expressed as lipid droplet content per cell, rather than normalized to cell size. This approach, based on single-cell analysis with sufficient sampling, minimized potential bias due to cell size heterogeneity.

### Lipid droplet fluorescence staining

To visualize intracellular neutral lipids, we employed BODIPY 493/503, a hydrophobic dye known for its specificity to lipid droplets. This dye was sourced from Invitrogen (item number D3922) and was prepared by dissolving in dimethyl sulfoxide (DMSO) to make a 1 mM stock solution, which was further diluted to a working concentration of 1 μM immediately before use. For the staining procedure, approximately 1×10^5^ cells were seeded into specialized dishes suitable for confocal microscopy. After subjecting the cells to various treatments such as irradiation or exposure to specific inhibitors, BODIPY 493/503 and Hoechst 33342 were added to the culture. The cells were incubated at 37 °C for 30 minutes to allow for adequate staining of lipids and nuclei. Post-incubation, cells were imaged using a confocal microscope to assess the distribution and abundance of lipid droplets.

### Cell proliferation assay

Cell proliferation was quantified using the crystal violet staining method. Cells were seeded at a density of 5×10^4^ cells per well in 6-well plates and allowed to adhere overnight. After treatment under various experimental conditions, cells were fixed with 4% formaldehyde and stained with 0.1% crystal violet. Excess stain was washed away with distilled water, and the dye retained by the cells was solubilized with 30% acetic acid. The absorbance was measured at 595 nm using a microplate reader to quantify cell proliferation.

### Cell clone formation experiment

For clonogenic assays, cells were plated at low density in 6-well plates and allowed to grow until visible colonies formed. After treatment, cells were incubated for 14 days. Colonies were fixed with ethanol and stained with 0.5% crystal violet. Colonies consisting of more than 50 cells were counted under a light microscope. The plating efficiency and surviving fraction were calculated to assess the clonogenic survival.

### Statistics

All experiments were independently repeated at least three times and all data are presented as the mean ± standard error. Student's *t*-tests were employed for statistical analysis, and probability (*p*) value less < 0.05 was considered statistically significant.

## Results

### ATMLP is highly expressed in NSCLC

**Figure 1:**
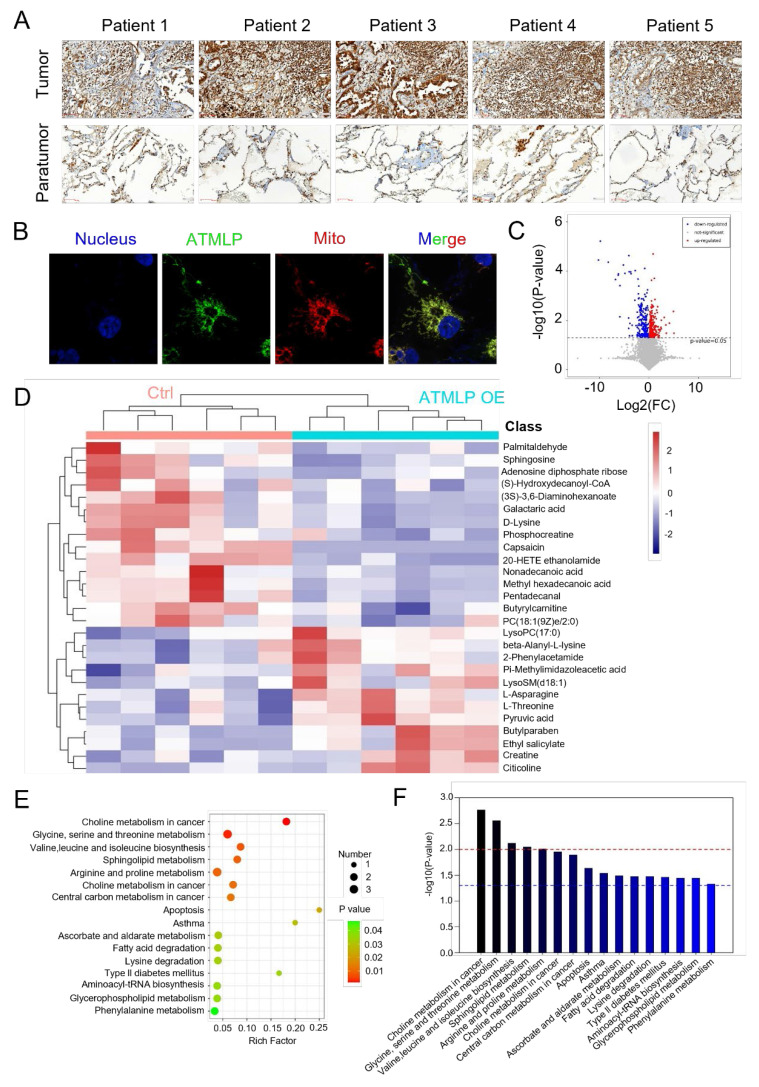
** ATMLP Is Highly Expressed in NSCLC.** (A) Representative IHC images of ATMLP expression in NSCLC tissues and corresponding paratumor tissues. (B) ATMLP GFP fusion protein was expressed in A549 cells as described. Mitochondria were stained by Mito-tracker Red CMXRos dye. Nucleus were stained by Hoechst33342. (C) Volcano plot of differential metabolites in untargeted metabolomics. A549 cells were transfected with ATMLP overexpression plasmid, and the cells were collected at 48 h for untargeted metabolomics assay. Red dots represent significantly up-regulated differential metabolites in the experimental group, blue dots represent significantly down-regulated differential metabolites, and gray dots represent differential metabolites that are not significant. (D) Differential metabolites were analyzed by horizontal and vertical clustering. Horizontal coordinates indicate sample names and vertical coordinates indicate differential metabolites. ATMLP OE is the overexpression group and Ctrl is the reference group. (E) Metabolic pathway bubble diagram. Vertical coordinates are metabolic pathway names; horizontal coordinates are enrichment factors (Rich factor). (F) KEGG metabolic pathway enrichment. The red line indicates a p-value of 0.01 and the blue line indicates a p-value of 0.05.

Our previous research has affirmed that ionizing radiation enhances adenine methylation at position 1313 of lncRNA AFAP1-AS1, initiating a non-cap-dependent translation to produce the 90-amino acid peptide ATMLP. While its role in autophagy regulation and tumorigenesis has been established, its function in tumor progression and treatment resistance remains unclear. To investigate ATMLP's clinical relevance, we performed immunohistochemical analysis on NSCLC tissues and matched adjacent non-tumorous tissues from 166 patients. ATMLP expression was significantly elevated in tumor tissues compared to controls (Figure 1A). Consistent with prior findings, ATMLP was localized to mitochondria in A549 lung cancer cells, further supporting its involvement in NSCLC biology (Figure 1B).

Mitochondria, as the central hub of cellular metabolism, play a critical role in energy production and biosynthetic processes 10. Given ATMLP's mitochondrial localization, we investigated its effects on mitochondrial metabolism using untargeted metabolomics. A549 cells were transfected with either an ATMLP overexpression plasmid or a control, followed by comprehensive metabolic profiling. Volcano plots revealed substantial metabolic shifts, with 262 metabolites significantly downregulated and 309 upregulated in response to ATMLP overexpression (Figure 1C). In order to more intuitively show the relationship between samples and the expression differences of metabolites among different samples, we performed Hierarchical Clustering on the expression of all significant differential metabolites and the top 50 differential metabolites with VIP values (Figure 1D). Pathway enrichment analysis using KEGG identifiers indicated significant alterations in lipid and glucose metabolism pathways, illustrated by an enrichment plot (Figure 1E-F). These findings underscore ATMLP's critical role in modulating mitochondrial metabolism. Its overexpression in NSCLC tissues suggests a functional link to the metabolic reprogramming essential for tumor survival and progression.

### ATMLP regulates lipid metabolism

**Figure 2:**
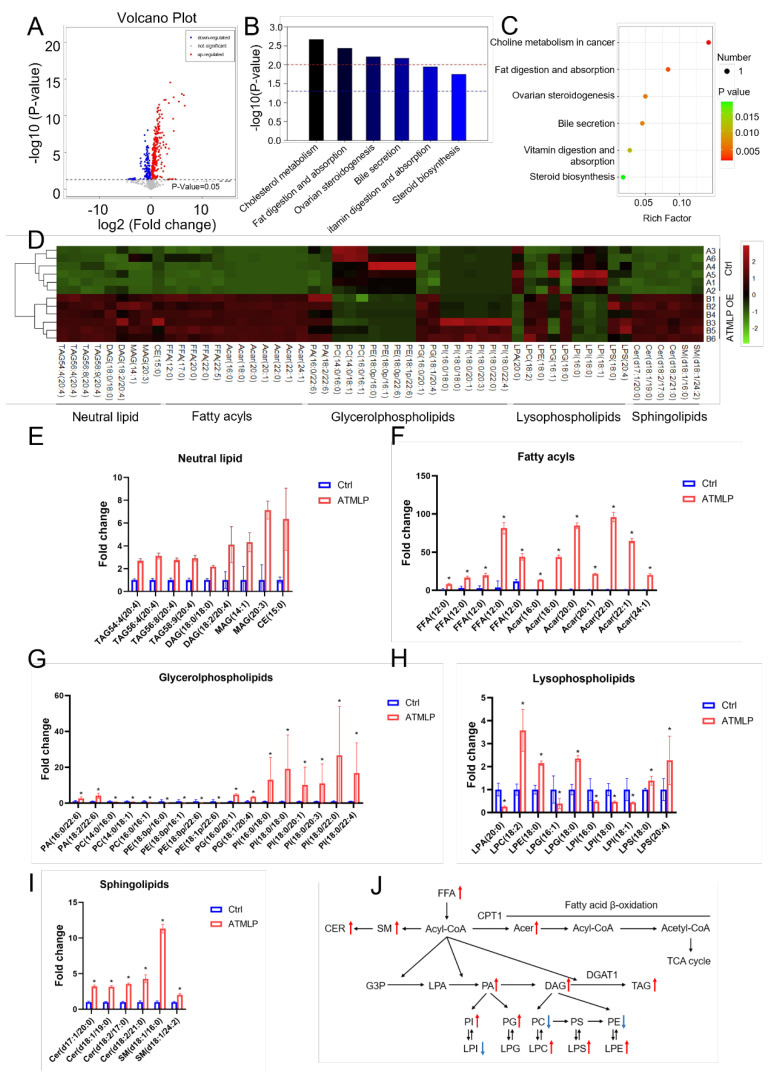
** ATMLP Regulates Lipid Metabolism.** (A) Volcano plot of differential metabolites in targeted metabolomics. A549 cells were transfected with ATMLP overexpression plasmid, and the cells were collected at 48 hours for lipid-targeted metabolomics. (B) KEGG metabolic pathway enrichment. (C) Metabolic pathway bubble diagram. Specifically, as described in Figure 1. (D) Heat map of differential metabolites. Each set has 6 parallel samples. (E-I) Differential metabolite analysis, * represents p < 0.05. 6 parallel samples were included in each set. (J) Network diagram of differential metabolites in lipid metabolism. Where red arrows represent metabolites increased upon overexpression of ATMLP, and blue arrows represent decreased. Ctrl represents the control group and ATMLP represents the overexpression group.

Building on findings from untargeted metabolomics, we observed that ATMLP overexpression led to the most significant changes in lipid metabolites. To investigate the specific role of ATMLP in lipid metabolism regulation, we performed a detailed lipidomic analysis in A549 cells. Cells were transfected with either an ATMLP overexpression plasmid or a control plasmid, and lipid profiles were assessed 48 hours post-transfection. Volcano plot analysis identified 141 downregulated and 326 upregulated lipid metabolites in the ATMLP-overexpressing cells (Figure 2A). Pathway enrichment analysis, based on KEGG identifiers, revealed that ATMLP influenced key lipid-related pathways, including cholesterol metabolism, fat digestion and absorption, ovarian steroidogenesis, bile secretion, vitamin digestion and absorption, and steroid biosynthesis (Figure 2B and C). Further statistical categorization and analysis of the most significantly altered metabolites revealed a marked increase in neutral lipid species, including triacylglycerols (TAG), diacylglycerols (DAG), monoacylglycerols (MAG), and cholesteryl esters (CE) (Figures 2D-I). A schematic representation of lipid dynamics indicated that ATMLP overexpression predominantly promoted the accumulation of these neutral lipids (Figure 2J). These findings demonstrate that ATMLP profoundly modulates cellular lipid metabolism, particularly enhancing the accumulation of neutral lipid components. This lipid metabolic reprogramming could play a crucial role in supporting NSCLC cell survival under therapeutic stress.

### ATMLP promotes the accumulation of intracellular lipid droplet

**Figure 3:**
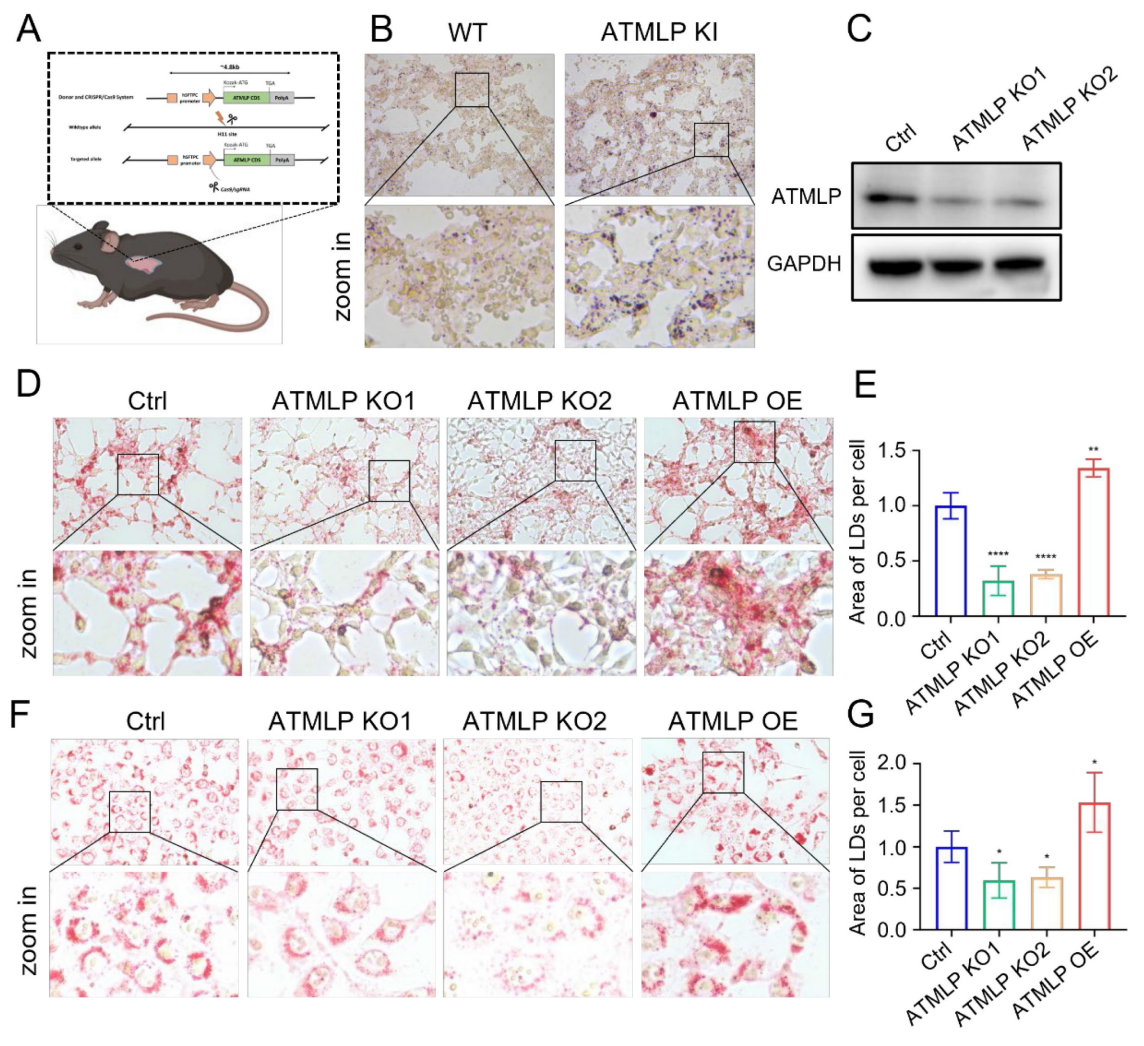
** ATMLP Promotes the Accumulation of Intracellular Lipid Droplet.** (A) Pattern of C57BL/6JGpt mice specifically expressing ATMLP in lung epithelial cells. (B) Lung tissues of ATMLP wild-type (WT) and lung epithelial cell ATMLP-specific knock-in (ATMLP KI) mice were stained with oil red. Lipid droplet content was assessed by oil red staining of adult mouse lung tissues after fixation by formalin and frozen sections. Enlarged images are shown below. (C) Expression levels of ATMLP in knock out cell lines (A549). GAPDH was used as an internal reference. The experiments were all repeated at least three times. (D and F) Lung cancer cells stained with oil red. Where Ctrl is the control group, ATMLP KO is the knockout group and ATMLP OE is the overexpression group. Shots were taken using a 40x objective, 10x eyepiece. (E and G) Statistical analysis of graphs D (A549) and F (H460), respectively. * p < 0.05, ** p < 0.01, and **** p < 0.0001.

LD are critical organelles in cellular lipid metabolism, serving as primary sites for the storage of neutral lipids and playing pivotal roles in metabolic processes such as lipid synthesis and mobilization 11. To investigate the effects of ATMLP on LD accumulation, we utilized a genetically engineered mouse model with lung epithelial cell-specific ATMLP overexpression in C57BL/6JGpt mice 9. Lung tissues from ATMLP knock-in and wild-type mice were examined, revealing significantly elevated LD content in the knock-in mice as determined by oil red staining (Figure 3A-B).

To further confirm ATMLP's role in promoting LD accumulation, we employed a non-toxic, genetically engineered Salmonella typhimurium strain carrying an ATMLP overexpression vector 12, injected into C57BL/6 mice via tail vein. Four days post-injection, liver tissue analysis showed markedly increased LD content in ATMLP-overexpressing mice, with oil red staining displaying intense and widespread lipid accumulation compared to controls (Figure S1A). Concurrent H&E staining revealed structural changes and prominent vacuoles in the liver tissue of ATMLP-overexpressing mice, indicative of significant lipid deposition. Biochemical analysis corroborated these findings, showing elevated triglyceride (Figure S1B) and cholesterol levels (Figure S1C) in the liver. To extend these findings, ATMLP knockout (KO) and overexpression (OE) cell lines were established across various lung normal and cancer cell types. Western blot validation confirmed that ATMLP knockout was highly efficient (Figure 3C). *In vitro* analyses using oil red and BODIPY 493/503 staining demonstrated that ATMLP overexpression significantly enhanced intracellular LD accumulation, while ATMLP knockout resulted in reduced LD content (Figure 3D-G, Figure S1D-F). These results validate ATMLP's role in promoting LD formation, highlighting its potential impact on lipid metabolism and tumor cell adaptation.

### Ionizing radiation promotes lipid droplets accumulation by inducing ATMLP expression

**Figure 4:**
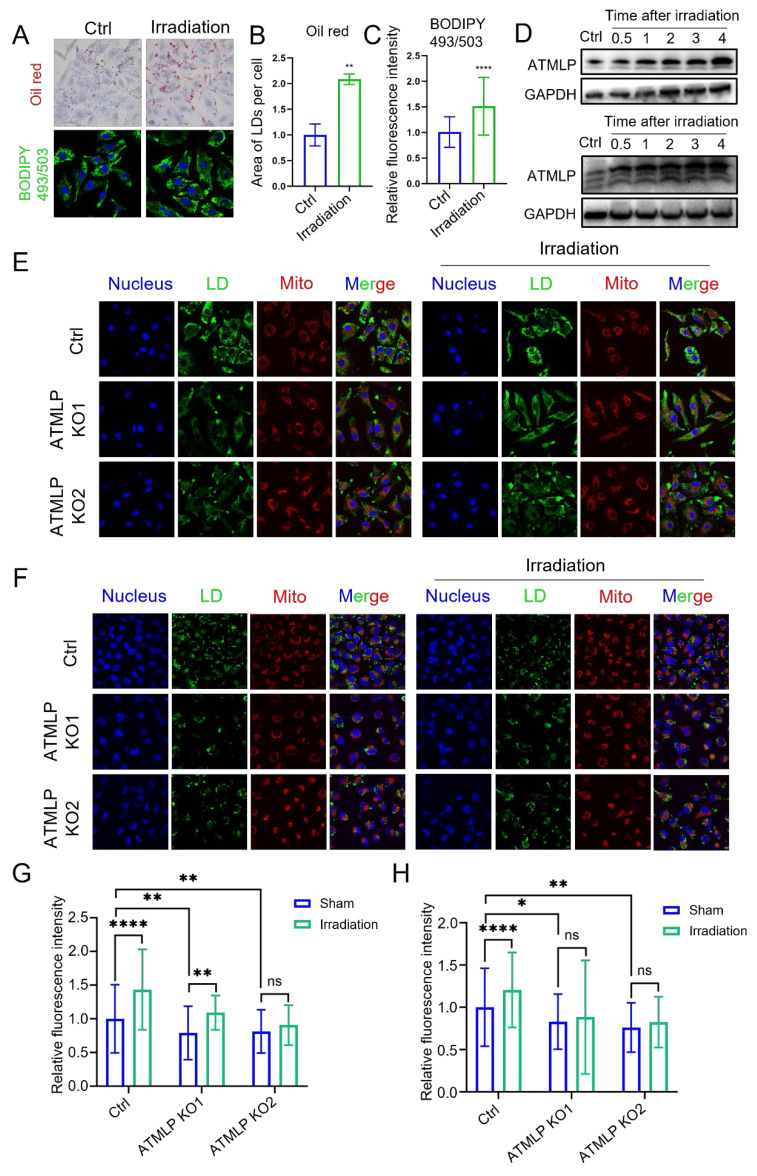
** Ionizing Radiation Promotes Lipid Droplets Accumulation by Inducing ATMLP Expression.** (A) LD staining verified the effect of irradiation on lipid droplets. Two methods were validated using oil red staining (upper panel) and BODIPY 493/503 fluorescent probe (lower panel), respectively. A549 cells were irradiated with 4Gy X-rays 24 hours after inoculation and stained at 48 hours. (B and C) The two staining results were analyzed and counted separately by imageJ software. The experiments were all repeated three times. ** p < 0.01, and **** p < 0.0001. (D) Expression levels of ATMLP in two different lung cancer cell lines (A549 and H1299) 4 days after irradiation. GAPDH was used as an internal reference. Cells were irradiated with 2 Gy X-rays. The experiments were all repeated three times. (E and F) Schematic representation of LD staining of two lung cancer cell lines. BODIPY 493/503 (lipid droplets, green), Mito-tracker Red CMXRos (mitochondria, red) and Hoechst33342 (nucleus, blue) staining of A549 cells (E) and H1299 cells (F) 48 hours after X-ray irradiation. Shots were taken at 60× objective and 10× eyepiece. (G and H) Statistical results of LDs staining in A549 cells (E) and H1299 cells (F), respectively. Ctrl is the control group and ATMLP KO is the knockout group. The experiments were all repeated three times. ns non-significant, * p < 0.05, ** p < 0.01, and **** p < 0.0001.

In our previous study, we demonstrated that ionizing radiation initiates ATMLP translation by enhancing adenine methylation at the 1313 locus of lncRNA AFAP1-AS1 13. Additionally, radiation is known to disrupt lipid metabolism, leading to LD accumulation. However, the relationship between these phenomena remains unclear. To explore this, A549 cells were subjected to 2 Gy X-ray irradiation, and LD accumulation was assessed. Both oil red and BODIPY 493/503 staining revealed a significant post-irradiation increase in LD content (Figure 4A-C). Concurrently, Western blot analysis of ATMLP protein levels across three NSCLC cell lines demonstrated an increase in ATMLP expression following radiation (Figure 4D). Further experiments using A549 and H1299 cells showed that ATMLP knockout effectively attenuated radiation-induced LD accumulation, as evidenced by reduced LD staining in irradiated knockout cells compared to controls (Figure 4E-H). These findings underscore the pivotal role of ATMLP in mediating LD accumulation in response to ionizing radiation. By linking radiation-induced ATMLP upregulation to alterations in lipid metabolism, this study highlights a potential mechanism underlying radioresistance in NSCLC.

### Increased radioresistance in NSCLC is mediated by ATMLP through lipid droplets accumulation

Metabolic reprogramming, a hallmark of cancer, is closely tied to tumor aggressiveness and resistance to therapy, with LD emerging as critical markers of these traits 14,15. Given that ionizing radiation induces ATMLP expression, which subsequently promotes LD accumulation, we hypothesized that this pathway underlies the radioresistance observed in NSCLC.

**Figure 5:**
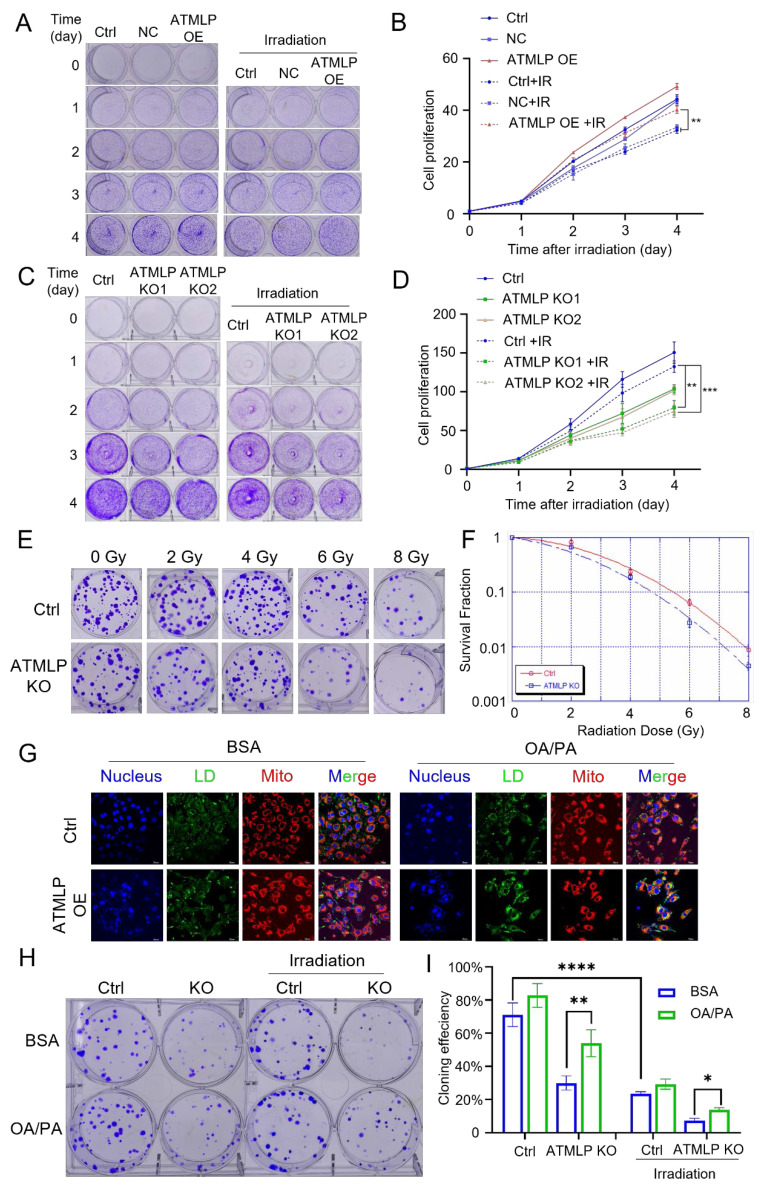
** Increased Radioresistance in NSCLC Is Mediated by ATMLP Through Lipid Droplets Accumulation.** (A-D) Effect of ATMLP on lung epithelial cell proliferation. 4×104 HSAEC1-KT (A) and H1299 (B) cells were inoculated into 6-well plates and subjected to 2 Gy X-ray irradiation after 24 h. The cells were fixed and stained with crystal violet after 0, 1, 2, 3, and 4 days, respectively. B and D are the statistical results of A and D, respectively. The experiments were all repeated three times. Ctrl is the control group; NC is the negative control group; ATMLP OE is the overexpression group; ATMLP KO is the knockout group. IR is 2 Gy X-ray irradiation. (E) Effect of ATMLP on the rate of cell clone formation. A549 cells were fixed 14 days after irradiation with 0, 2, 4, 6, 8 Gy X-rays and stained with crystal violet. (F) Statistical results of clone formation experiments. The number of cell clones was counted and analyzed, and the cloning efficiency was calculated. (G) Lipid droplet fluorescence staining is representative of the figure. A549 cells were inoculated into microscope-specific petri dishes with BSA and OA/PA mixtures for 48 hr, respectively, and stained for LDs and mitochondria by BODIPY 493/503 fluorescent probes and Mito-tracker Red CMXRos, respectively. (H) A549 cells were treated with BSA (control) and OA/PA mixture (high lipid droplets group) for 24 h after inoculation and then subjected to 2 Gy X-ray irradiation, fixed after 14 days and stained with crystal violet. 80 cells were inoculated in the Ctrl group and 150 cells in the IR group. All experiments were repeated three times independently. (I) Statistical results of Figure 5H. * represents p < 0.05; ** represents p < 0.01; **** represents p < 0.0001.

To investigate, we evaluated the proliferation of lung epithelial cells with ATMLP overexpression. These cells displayed significantly enhanced proliferation rates compared to controls. Upon exposure to 2 Gy X-rays, all cells exhibited reduced growth; however, ATMLP-overexpressing cells demonstrated significantly less growth inhibition (Figure 5A and B). Further assessment using H1299 and A549 cell lines revealed that ATMLP knockout markedly increased radiosensitivity, evidenced by decreased proliferation and survival post-irradiation (Figure 5C-D, Figure S2A). Consistent findings were observed in A549 clone formation assays, where ATMLP-deficient cells exhibited reduced clonogenic survival following radiation (Figure 5E-F). To establish the connection between LD and radioresistance, ATMLP knockout cells were supplemented with oleic acid and palmitic acid to artificially restore LD levels (Figure 5G, Figure S2B). Clone formation assays showed that this intervention partially rescued the radiosensitivity of ATMLP-deficient cells, improving survival after 2 Gy X-ray exposure (Figure 5H-I). These results highlight the pivotal role of ATMLP in enhancing radioresistance by modulating LD dynamics, offering insights into potential therapeutic strategies targeting metabolic pathways to improve radiotherapy outcomes in NSCLC.

### ATMLP promotes lipid droplets accumulation and radioresistance through activation of the AKT pathway

**Figure 6:**
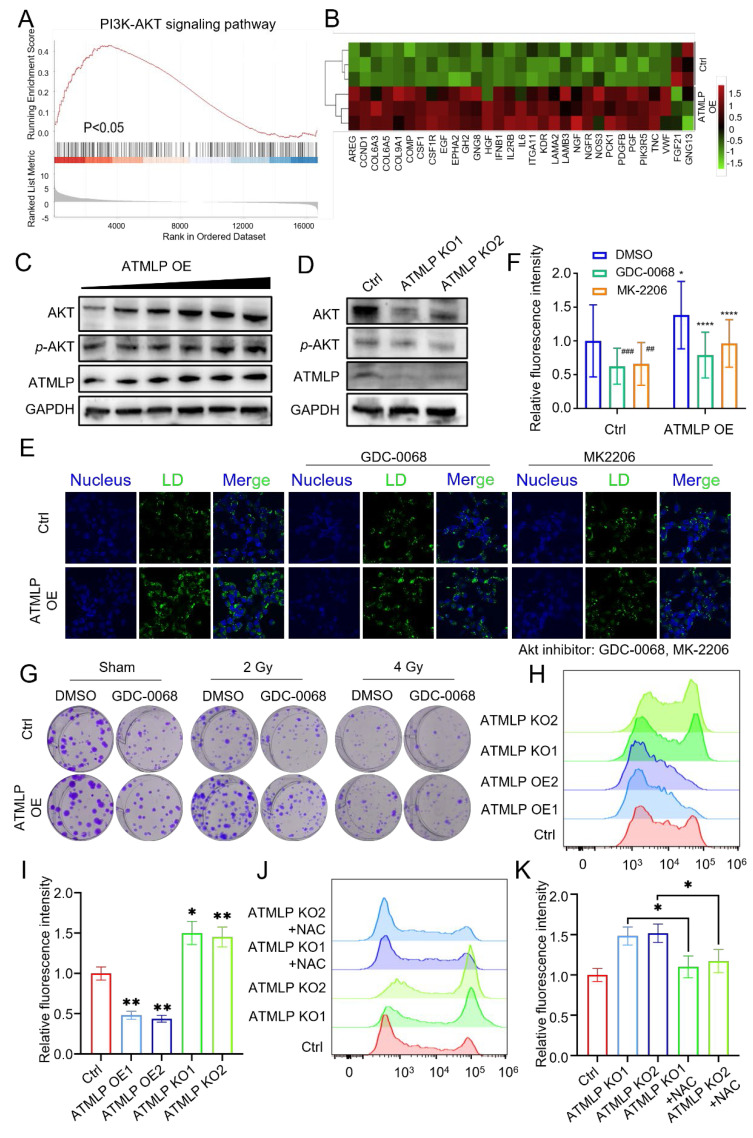
ATMLP Promotes Lipid Droplets Accumulation and Radioresistance Through Activation of the AKT Pathway. (A) Transcriptomics sequencing of differentially expressed genes for GSEA analysis. (B) Heatmap of PI3K-AKT pathway-related differential genes. (C and D) Western Blot assay to detect AKT, phosphorylated AKT (p-AKT) protein expression. GAPDH as an internal reference. (E and F) 1×10^5^ H1299 cells were inoculated into microscope-specific cell culture dishes, ATMLP overexpressing adenovirus was added 24 h later, 2 μM GDC0068 and MK2206 were added 24 h later, and 24 h later, lipid droplets staining was performed by BODIPY493/503 probe, and photographs were taken for fluorescence confocal microscopy observation. Quantitative analysis was performed by ImageJ software. Quantitative analysis was performed by ImageJ software. * p < 0.05; ** p < 0.01; *** p < 0.001; **** p < 0.0001. (I) Different numbers of A549 cells were inoculated into 6-well plates, AKT inhibitor (2 μM GDC-0068) was added to the medium, which was subjected to X-ray irradiation after 24 hours and continued to be cultured for 14 days and then stained with crystal violet. The experiments were all repeated three times. (H) Flow cytometry was used to detect ROS levels in H1299 cells. Cells were seeded into a 6-well plate and cultured to the exponential growth phase. The cells were then incubated with DCFH for 30 minutes, after which reactive oxygen species levels were quantified using Flow cytometry. (I) Statistical analysis of Figure H. The mean fluorescence intensity per cell was calculated and normalized to the control group. NAC (N-acetylcysteine) was used at a working concentration of 5 mM to reduce intracellular ROS levels. (J and K) The same experimental procedures were applied as those described for Figures H and I.

To uncover the molecular mechanisms by which ATMLP influences LD accumulation and contributes to radioresistance, transcriptomic sequencing was performed. The analysis identified significant transcriptional changes: 943 genes were upregulated, while 174 were downregulated (|fold change| ≥ 1.5, p < 0.05), as visualized in a volcano plot (Figure S3A). Gene Ontology (GO) analysis linked these differentially expressed genes to key biological processes and pathways, including arachidonic acid metabolism, the PI3K/AKT signaling pathway, the FoxO signaling pathway, and the MAPK signaling pathway, all of which intersect with lipid metabolism and tumor progression (Figure S3B). Gene Set Enrichment Analysis (GSEA) further emphasized the upregulation of PI3K/AKT pathway-related genes such as PIK3R5, PCK1, and KDR in ATMLP-overexpressing cells (Figures 6A-B). Western blot analysis corroborated these findings at the protein level, showing increased ATMLP, AKT, and phosphorylated AKT (p-AKT) in ATMLP-overexpressing cells, indicative of AKT pathway activation (Figure 6C). Conversely, ATMLP knockout significantly reduced p-AKT levels (Figure 6D).

To further dissect the AKT pathway's role in LD dynamics, we employed insulin to activate AKT and inhibitors Ipatasertib (GDC-0068) and MK-2206 to suppress it. In H1299 cells, ATMLP overexpression induced LD accumulation, which was markedly reduced upon AKT inhibition (Figures 6E and F). Conversely, AKT activation in ATMLP-deficient cells restored LD levels, underscoring the regulatory role of AKT in mediating ATMLP-driven lipid changes (Figures S4A and B). These results were consistent in A549 cells and corroborated by oil red and BODIPY 493/503 staining (Figures S4C-H). Finally, the functional consequences of AKT pathway activation were validated in clone formation assays following irradiation. A549 cells treated with AKT inhibitors exhibited significantly reduced clonogenic survival post-irradiation, demonstrating the critical role of the AKT pathway in conferring ATMLP-mediated radioresistance (Figure 6G). These findings were further supported by proliferation assays in A549 cells (Figure S5A). Collectively, these results establish that ATMLP promotes LD accumulation and enhances radioresistance in NSCLC cells through activation of the AKT pathway, highlighting potential therapeutic targets to improve radiotherapy efficacy.

In addition, we observed that ATMLP knockout cells exhibited excessive ROS production, which markedly inhibited AKT phosphorylation. Conversely, radiation-induced ATMLP expression significantly reduced intracellular ROS levels, thereby sustaining AKT activation (Figure 6H-K, Figure S5B). These findings suggest that ATMLP promotes AKT signaling not by direct binding to upstream regulators, but through the regulation of oxidative stress, establishing a ROS-dependent mechanism that enhances radioresistance.

### Inhibition of ATMLP and AKT expression increases the radiosensitivity of NSCLC *in vivo*

**Figure 7:**
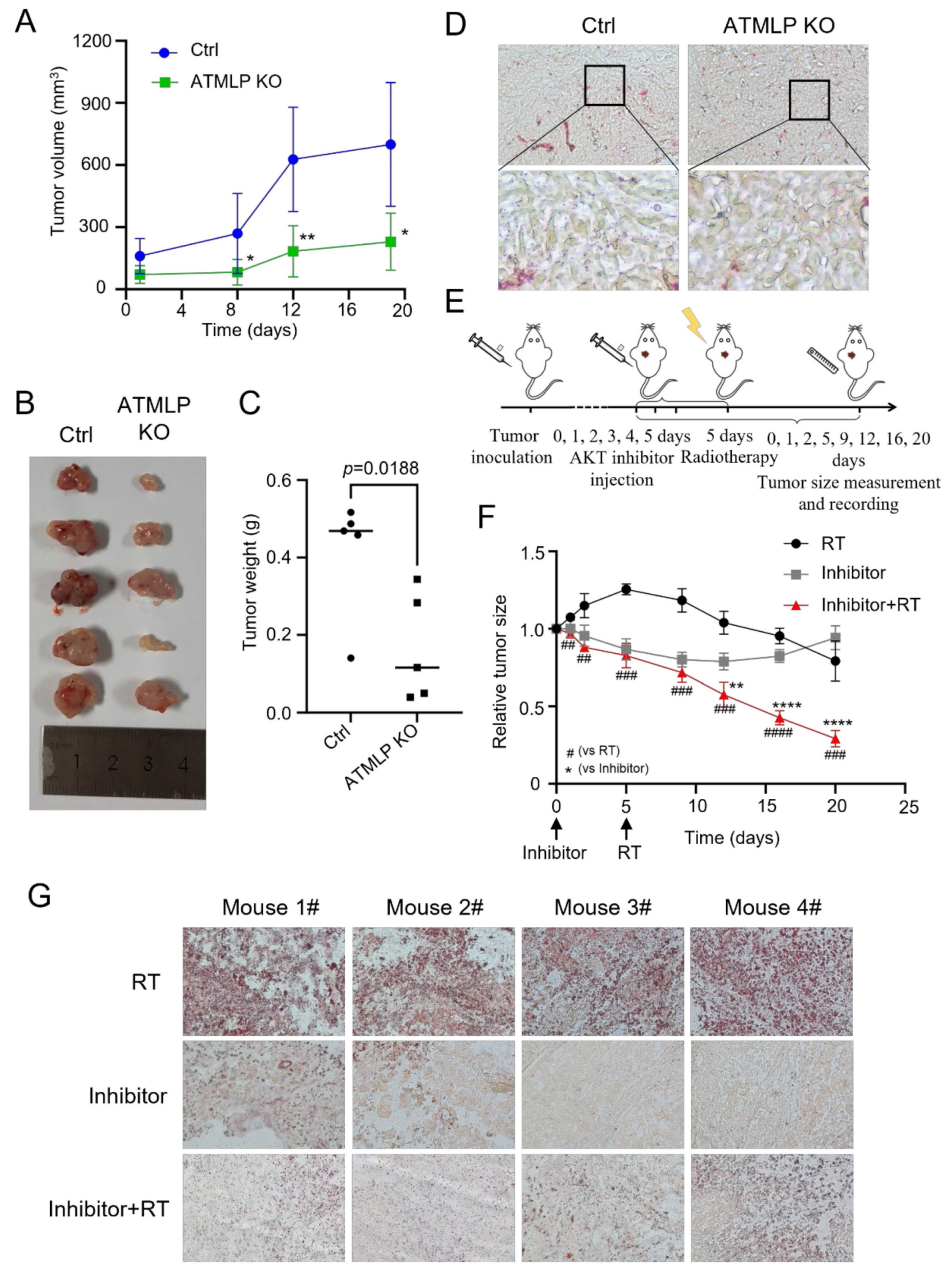
Inhibition of ATMLP and AKT Expression Increases the Radiosensitivity of NSCLC *In Vivo*. (A) A xenograft model was established by subcutaneous injection of 1×10^6^ A549 cells into the buttocks of 8-week-old nude mice (n=5). Tumor size was observed and recorded. The horizontal and vertical markers are the time after cell inoculation, and the vertical coordinate is the tumor volume. (B) The mice were dissected at week 19, and the tumors were peeled out and photographed. (C) Mice were dissected at week 19, and the tumors were peeled out and weighed. (D) The xenografts were fixed and frozen sectioned, and the lipid droplet content was assessed by oil red staining. The figure below shows a local magnification. (E) Schematic diagram of the treatment of xenograft tumor model. As described previously, xenograft tumors were established using A549 cells, and after the tumors grew to the appropriate size, they were injected with AKT inhibitor (GDC-0068) peri-tumorally for 6 consecutive days, and then treated with 4Gy X-rays, and the growth of the tumors was continuously observed and recorded. (F) Changes in xenograft volume in AKT inhibitor, radiation therapy and combined treatment mice. The experimental protocol was as described in D. (G) On the 20th day of graft treatment, mice were euthanized, tumors were excised, and frozen sections were stained with oil red. All animal experiments were approved by the Institutional Review Board (IRB) and the Animal Care and Use Committee (ACUC) of Soochow University, and were conducted in the SPF Animal Laboratory of Soochow University, in accordance with the ethical guidelines for animal welfare.

To investigate the physiological relevance of ATMLP and its clinical implications, we utilized a nude mouse xenograft model. A549 cells, including ATMLP KO and control lines, were subcutaneously injected into nude mice. Tumor growth in the ATMLP KO group was significantly slower than in controls, with marked reductions in tumor size and weight at 19 weeks post-injection (Figures 7A-C). Oil red staining of excised tumors revealed a substantial decrease in LD accumulation in ATMLP KO tumors, consistent with our *in vitro* findings of reduced lipid metabolism (Figure 7D).

To further explore therapeutic strategies, we combined AKT pathway inhibition with radiation therapy. Xenografts were treated with the AKT inhibitor Ipatasertib for six days, followed by 4 Gy X-ray irradiation. This combination therapy significantly suppressed tumor growth compared to either treatment alone, highlighting the synergistic effects of AKT inhibition and radiotherapy (Figure 7E). Tumor tissue analysis post-treatment demonstrated that AKT inhibition effectively reduced LD accumulation, a common response to radiation therapy, as confirmed by oil red staining (Figure 7F). To further validate the generalizability of our *in vivo* findings, we repeated the xenograft experiments using H1299 cells and obtained results consistent with those observed in the A549 model (Fig. S6).

These *in vivo* findings emphasize the therapeutic potential of targeting ATMLP and the AKT signaling pathway to enhance radiosensitivity in NSCLC. The results suggest that disrupting this pathway mitigates tumor-promoting metabolic adaptations, reduces tumor growth, and improves the efficacy of radiotherapy. This strategy provides a promising approach to address radioresistant phenotypes in NSCLC, paving the way for novel combination therapies in clinical settings.

## Discussion

**Figure 8:**
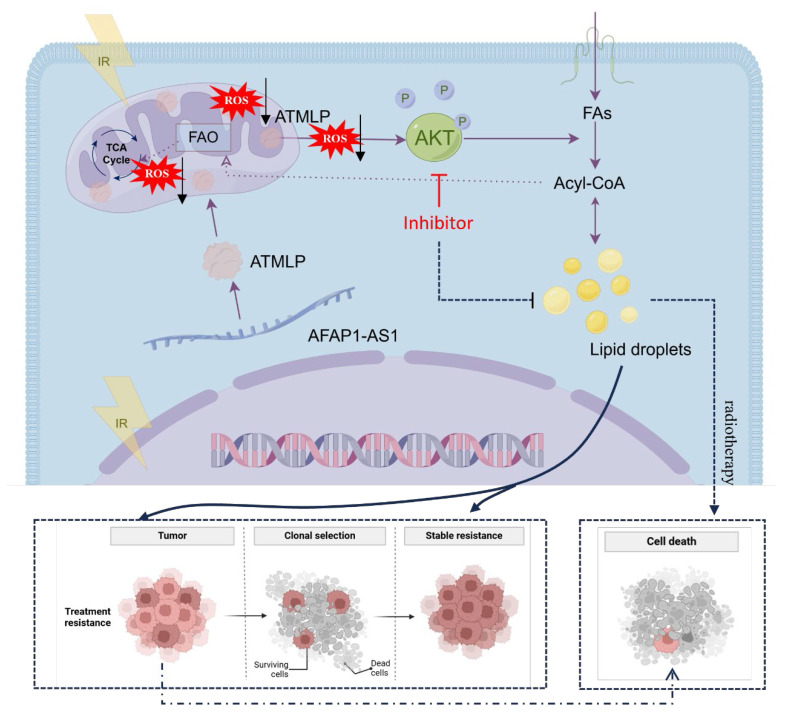
Working model for ATMLP function.

LncRNAs have emerged as critical regulators in cancer biology, influencing a myriad of cellular processes, including carcinogenesis and therapy resistance 16. Traditionally studied for their transcriptional and post-transcriptional regulatory roles, recent advances have unveiled their capacity to encode functional peptides and proteins that significantly impact cellular metabolism and disease progression 17,18. Notable examples include LINC00998-encoded SMIM30, which enhances proliferation and migration in hepatocellular carcinoma 19, and LINC00665-derived peptides that promote tumor invasion via pathways like CIP2A/PP2A and PI3K/AKT/NFκB 20. In our prior work, we identified ATMLP, a 90-amino-acid peptide encoded by lncRNA AFAP1-AS1, localized to mitochondria, which promotes tumorigenesis by modulating autophagy. In this study, we further demonstrate that ATMLP mediates radioresistance in NSCLC through reprogramming of lipid metabolism and activation of the AKT pathway. Mechanistically, ATMLP mitigates radiation-induced ROS accumulation, thereby preventing ROS-mediated inhibition of AKT phosphorylation and promoting intracellular lipid droplet formation. Given that ATMLP localizes to mitochondria whereas AKT activation predominantly occurs in the cytoplasm, a direct physical interaction between the two proteins is unlikely. Instead, the mitochondrial localization of ATMLP naturally points to its role in regulating mitochondrial function, with ROS serving as a key intermediary. Indeed, our data support this redox-mediated mechanism, showing that ATMLP deficiency leads to excessive ROS production, which in turn suppresses AKT signaling. On this basis, we focused on delineating the ROS-dependent ATMLP-AKT axis, rather than pursuing co-immunoprecipitation assays, as such approaches would be less informative for capturing this indirect mode of regulation. These findings highlight a dual role of ATMLP in regulating both ROS homeostasis and lipid metabolism to support tumor cell survival under radiative stress, underscoring its pivotal function in the adaptive response of NSCLC to radiotherapy (Figure 8). Notably, although supplementation with exogenous lipids (OA/PA) restored intracellular LD content in ATMLP-deficient cells, the rescue of radioresistance was only partial. This finding suggests that ATMLP may regulate radiosensitivity not solely through LD accumulation but also via additional mechanisms, such as redox homeostasis or other metabolic signaling pathways. These possibilities warrant further investigation in future studies.

Globally, lung cancer remains the leading cause of cancer-related mortality, with NSCLC comprising the majority of cases and often exhibiting intrinsic resistance to radiation therapy 1,21. This resistance underscores an urgent need for a deeper understanding of its underlying mechanisms to improve therapeutic outcomes 22,23. Metabolic reprogramming has long been recognized as a hallmark of cancer, enabling tumor cells to sustain growth and survive under stress conditions 11,24. Our findings highlight the centrality of LD in this reprogramming, with ATMLP facilitating LD accumulation as a cytoprotective mechanism. By activating the AKT pathway, ATMLP enhances the survival of NSCLC cells post-radiation, reinforcing the critical interplay between lipid metabolism and radioresistance. The role of LD in mediating resistance to oxidative stress has been well documented 25, but our study specifically identifies ATMLP as a key driver of this process in NSCLC. By promoting LD accumulation, ATMLP not only enhances tumor cell survival under radiative stress but also positions itself as a promising therapeutic target. Unlike prior studies that broadly address metabolic alterations, our work delineates a precise ATMLP-AKT-LD axis, providing a novel intervention point. Targeting ATMLP, either directly or through its downstream AKT signaling pathway, offers a potential strategy to mitigate LD-mediated radioresistance and sensitize NSCLC cells to radiation therapy. This aligns with emerging evidence supporting the utility of metabolic interventions to overcome therapeutic resistance 26. While our data demonstrate that ATMLP expression correlates with enhanced AKT activation, we did not detect direct physical interactions between ATMLP and AKT or its upstream regulators (e.g., PI3K, PTEN). Instead, our results indicate that ATMLP indirectly modulates AKT signaling through regulation of ROS levels. This highlights a novel mitochondria-ROS-AKT-lipid signaling axis in NSCLC radioresistance. Future studies employing co-immunoprecipitation or proximity ligation assays will be essential to clarify potential direct molecular interactions.

In the broader context of lncRNA-encoded peptides, several small peptides such as MOXI and SMIM30 have also been implicated in lipid metabolism and tumor progression. MOXI was reported to enhance mitochondrial fatty acid oxidation, while SMIM30 modulates phospholipid metabolism and oncogenic signaling. Unlike these peptides, ATMLP specifically promotes lipid droplet accumulation and radioresistance by modulating ROS—AKT signaling. This functional divergence highlights ATMLP's unique contribution to linking mitochondrial signaling, redox regulation, and lipid storage with therapeutic resistance in NSCLC. Consistent with our previous report 9, ATMLP was found to be upregulated in NSCLC tissues, where its high expression correlated with poor patient prognosis. Building on these findings, the present study focuses on elucidating the mechanistic basis of ATMLP-mediated radioresistance. Importantly, these data also suggest that ATMLP may serve as a potential predictive biomarker of radioresistance in NSCLC. To further establish its clinical relevance, we have initiated a prospective study to systematically evaluate ATMLP expression in tumor samples from NSCLC patients undergoing radiotherapy and to correlate it with treatment response and survival outcomes. Ultimately, validation of ATMLP as a biomarker could help stratify patients for personalized radiotherapy strategies and guide the development of targeted therapeutic interventions. In conclusion, our study expands the understanding of ATMLP's role in cancer metabolism and its contribution to radioresistance in NSCLC. By targeting the ATMLP-ROS-AKT-LD axis, there is potential to disrupt the metabolic adaptations that underlie resistance mechanisms, paving the way for more effective and personalized cancer treatments.

## Supplementary Material

Supplementary figures.
