# Supplementary material for: ATMLP Enhances Radioresistance in Non-Small Cell Lung Cancer through AKT-Mediated Lipid Droplet Accumulation
Source: Int J Biol Sci. 2026 Jan 21;22(4):1852–67. doi: 10.7150/ijbs.116401 (PMC12905571; doi:10.7150/ijbs.116401)
Supplement: Supplementary file 1 — Supplementary figures. [file ijbsv22p1852s1.pdf]

Supplementary data
SUP 1.

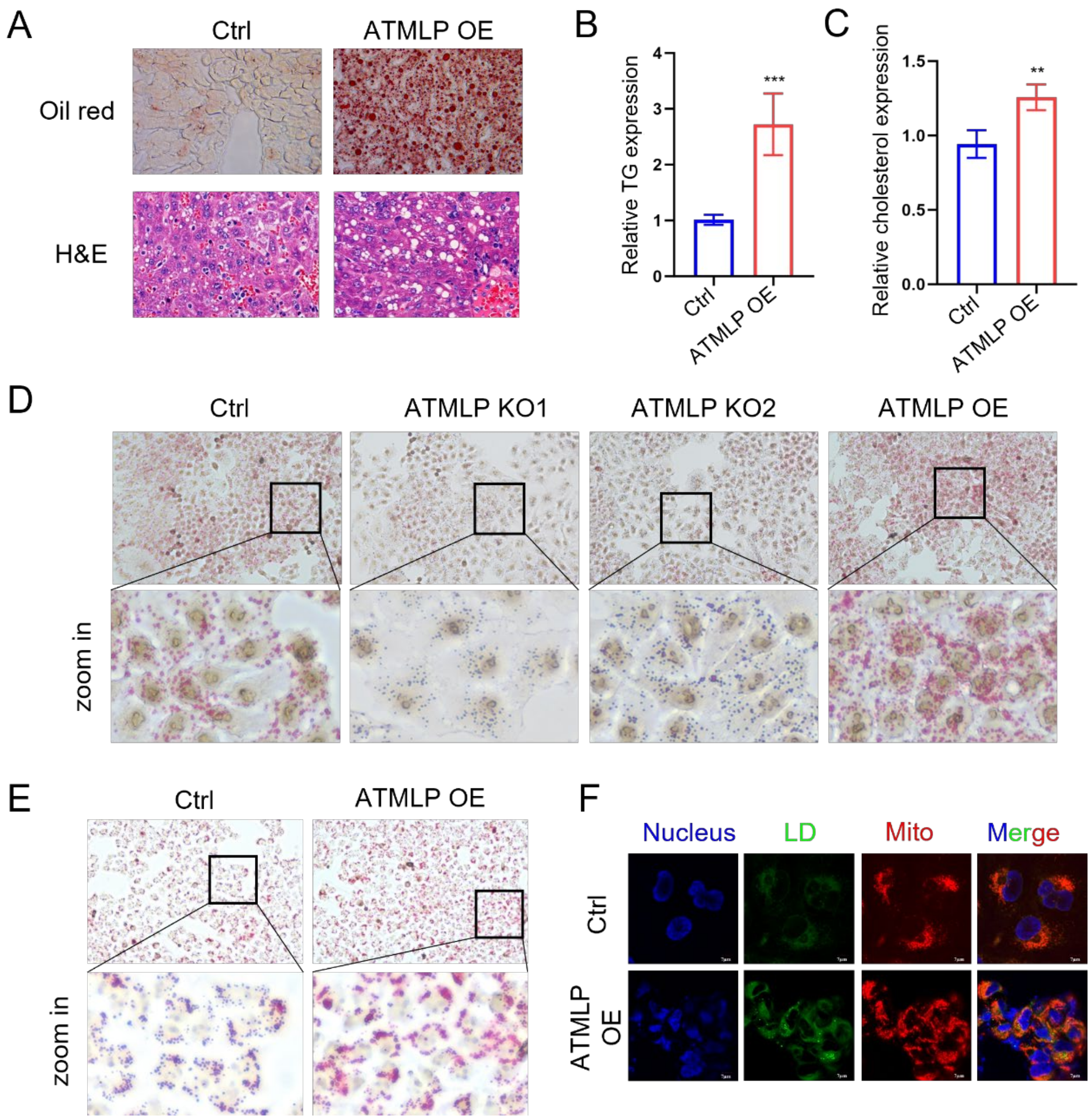

S1. (A) Oil red staining (top) and HE staining (bottom) of the livers of C57 mice. Salmonella was injected into C57 mice via tail vein, and the mice were executed 4 days later. Tissues were fixed with formalin and then frozen sectioned or paraffin sectioned. (B) is a mouse liver triglyceride (TG) content assay. (C) Mouse liver cholesterol content assay. ** $p<0.01$; *** $p<0.001$. (D) Hela cells stained with oil red. Where Ctrl is the control group, ATMLP KO is the knockout group and ATMLP OE is the overexpression group. Shots were taken using a 40x objective, 10x eyepiece. (E) L02 cells stained with oil red. (F) 1×105 Calu-1 cells were inoculated into microscope-specific cell culture dishes, and after the cells were stabilized, the lipid droplets were labeled with BODIPY 493/503 fluorescent probe.

SUP 2.

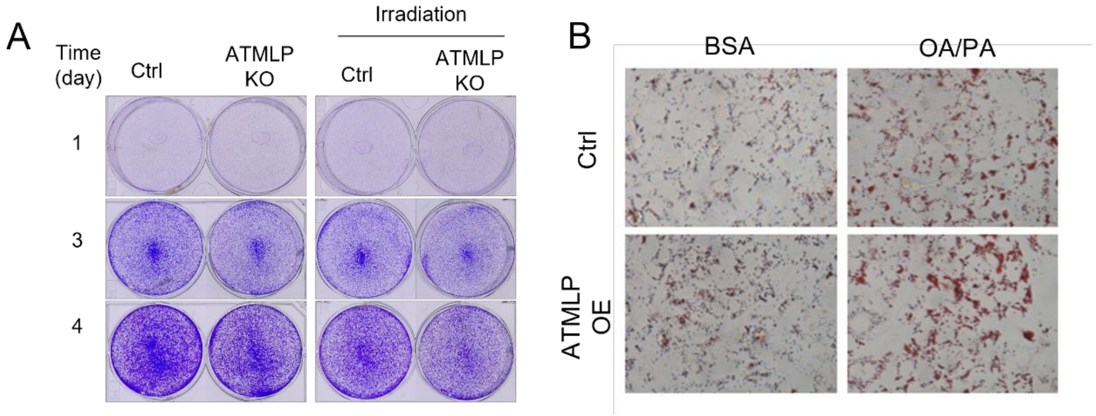

S2. **(A)** Effect of ATMLP on A549 cell proliferation. 4×10^4 A549 cells were inoculated into 6-well plates and subjected to 2 Gy X-ray irradiation after 24 h. The cells were fixed and stained with crystal violet after 1, 3, and 4 days, respectively. **(B)** Representative graph of oil red staining results. 1×10^5 A549 cells were inoculated into 6-well plates and treated with BSA or OA/PA mixture for 48 h. The intracellular lipid droplet content was assessed by oil red staining assay.

SUP 3.

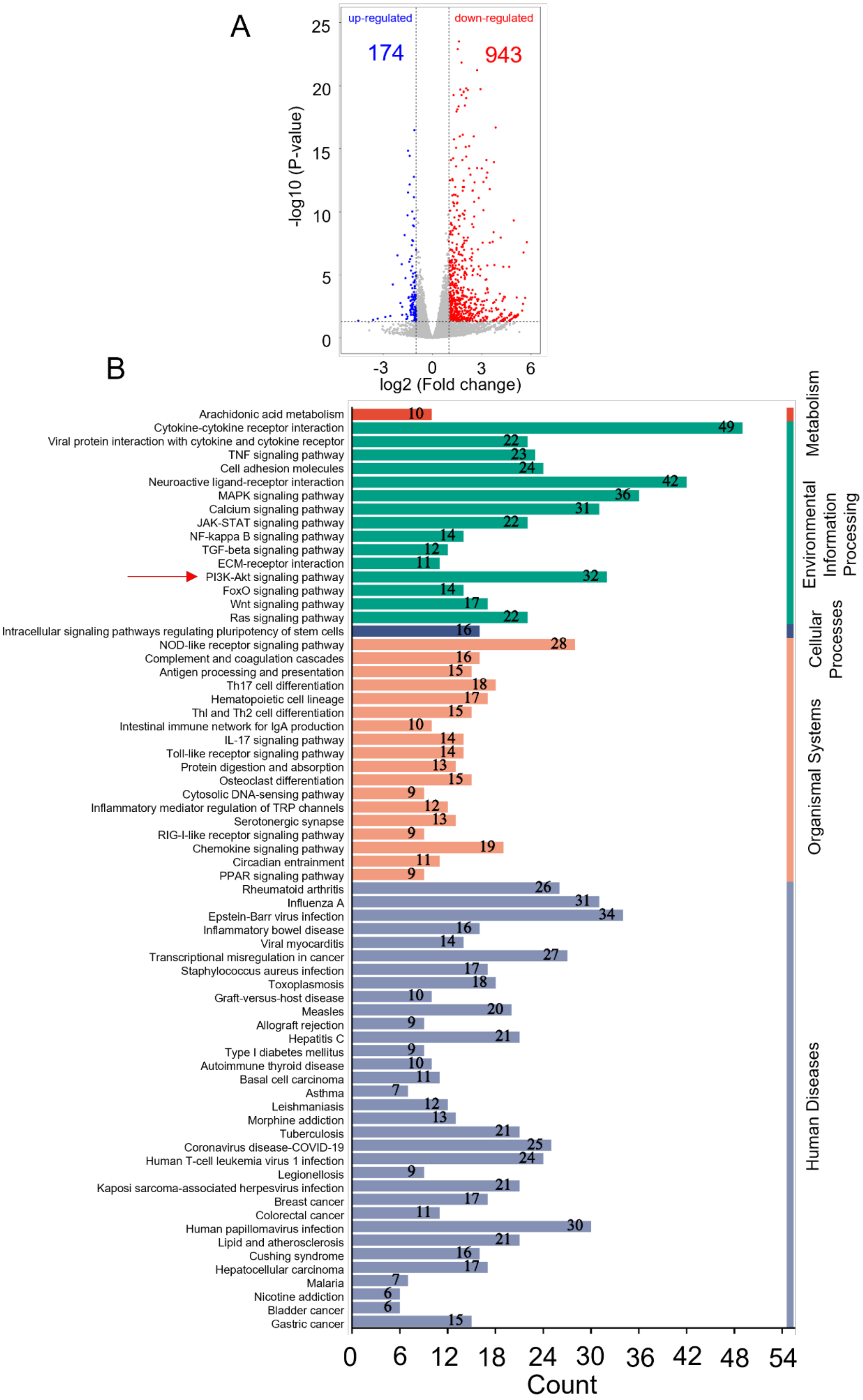

A
up-regulated
down-regulated
174
943
-log10 (P-value)
log2 (Fold change)
B
Arachidonic acid metabolism
Cytokine-cytokine receptor interaction
Viral protein interaction with cytokine and cytokine receptor
TNF signaling pathway
Cell adhesion molecules
Neuroactive ligand-receptor interaction
MAPK signaling pathway
Calcium signaling pathway
JAK-STAT signaling pathway
NF-kappa B signaling pathway
TGF-beta signaling pathway
ECM-receptor interaction
PI3K-Akt signaling pathway
FoxO signaling pathway
Wnt signaling pathway
Ras signaling pathway
Intracellular signaling pathways regulating pluripotency of stem cells
NOD-like receptor signaling pathway
Complement and coagulation cascades
Antigen processing and presentation
Th17 cell differentiation
Hematopoietic cell lineage
Thl and Th2 cell differentiation
Intestinal immune network for IgA production
IL-17 signaling pathway
Toll-like receptor signaling pathway
Protein digestion and absorption
Osteoclast differentiation
Cytosolic DNA-sensing pathway
Inflammatory mediator regulation of TRP channels
Serotonergic synapse
RIG-I-like receptor signaling pathway
Chemokine signaling pathway
Circadian entrainment
PPAR signaling pathway
Rheumatoid arthritis
Influenza A
Epstein-Barr virus infection
Inflammatory bowel disease
Viral myocarditis
Transcriptional misregulation in cancer
Staphylococcus aureus infection
Toxoplasmosis
Graft-versus-host disease
Measles
Allograft rejection
Hepatitis C
Type I diabetes mellitus
Autoimmune thyroid disease
Basal cell carcinoma
Asthma
Leishmaniasis
Morphine addiction
Tuberculosis
Coronavirus disease-COVID-19
Human T-cell leukemia virus 1 infection
Legionellosis
Kaposi sarcoma-associated herpesvirus infection
Breast cancer
Colorectal cancer
Human papillomavirus infection
Lipid and atherosclerosis
Cushing syndrome
Hepatocellular carcinoma
Malaria
Nicotine addiction
Bladder cancer
Gastric cancer
Metabolism
Environmental Information Processing
Cellular Processes
Organismal Systems
Human Diseases
Count

S3 (A) Transcriptomics sequencing of differential gene volcano maps that fulfill the conditions: Fold change≥1.5, p<0.5. **(B)** Differential gene pathway analysis.

SUP 4.

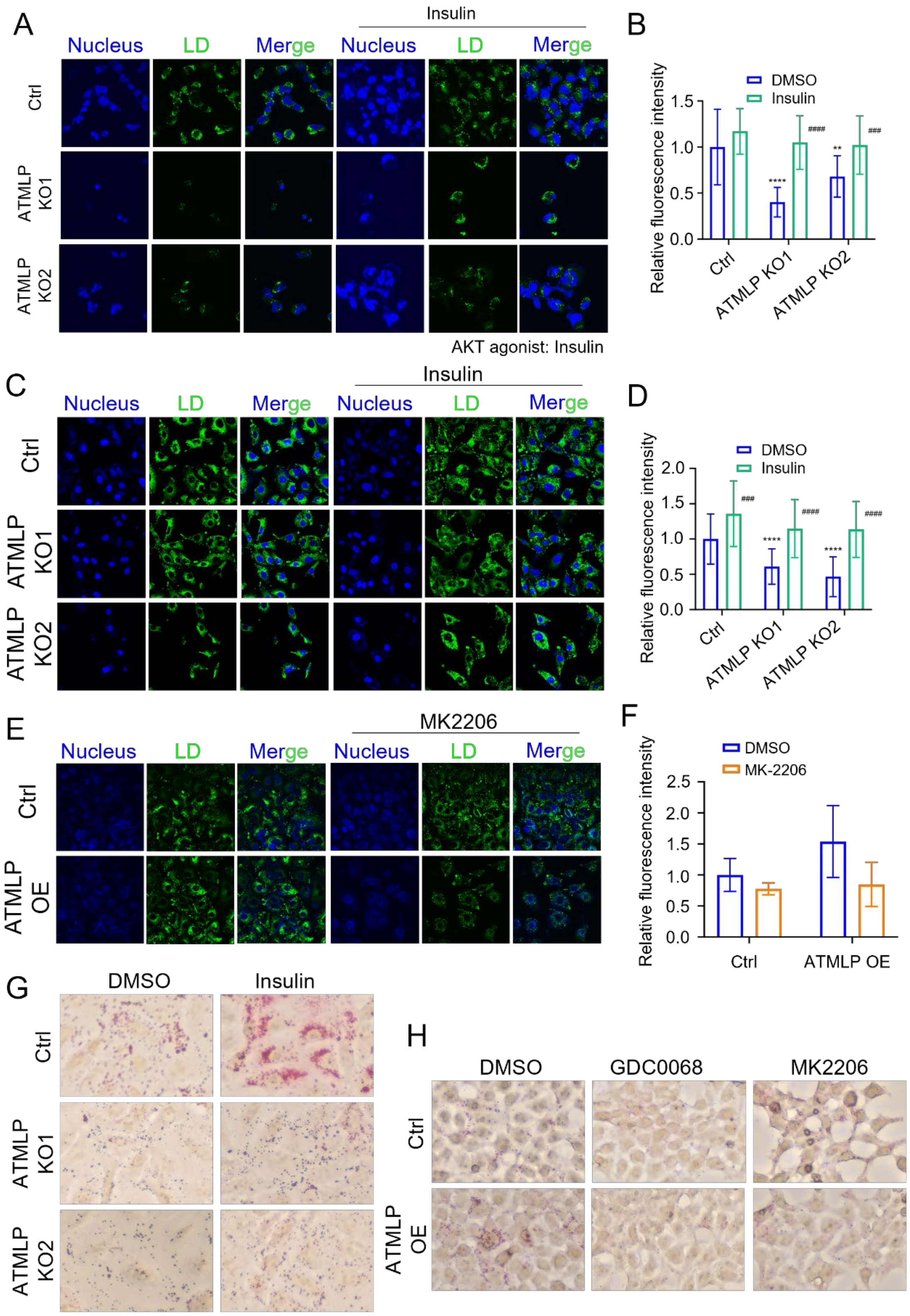

S4. **(A and B)** 1×10^5 H1299 cells were inoculated into microscope-specific cell culture dishes, 100 mU/mL of insulin was added after 24 h. After 24 h, the lipid droplets were stained by BODIPY493/503 probe and photographed by fluorescence confocal microscopy observation. Quantitative analysis was performed by ImageJ software. Quantitative analysis was performed by ImageJ software. * $p < 0.05$; ** $p < 0.01$; *** $p < 0.001$; **** $p < 0.0001$. **(C and D)** 1×10^5 A549 cells were inoculated into microscope-specific cell culture dishes, 100 mU/mL of insulin was added after 24 h. After 24 h, the lipid droplets were stained by BODIPY493/503 probe and photographed by fluorescence confocal microscopy observation. **(E and F)** 1×10^5 A549 cells were inoculated into microscope-specific cell culture dishes, ATMLP overexpressing adenovirus was added 24 hr later, 2 μM GDC0068 and MK2206 were added 24 hr later, and 24 hr later, lipid droplets staining was performed by BODIPY493/503 probe, and photographs were taken for fluorescence confocal microscopy observation. Quantitative analysis was performed by ImageJ software. Quantitative analysis was performed by ImageJ software. * $p < 0.05$; ** $p < 0.01$; *** $p < 0.001$; **** $p < 0.0001$. **(G)** 1×10^5 A549 cells were inoculated into 6-well plates cell culture dishes, 100 mU/mL of insulin was added after 24 h. After 24 h, the lipid droplets were stained by oil red staining. **(H)** 1×10^5 A549 cells were inoculated into 6-well plates cell culture dishes, ATMLP overexpressing adenovirus was added 24 hr later, 2 μM GDC0068 and MK2206 were added 24 hr later, and 24 hr later, lipid droplets staining was performed by oil red staining.

SUP 5.

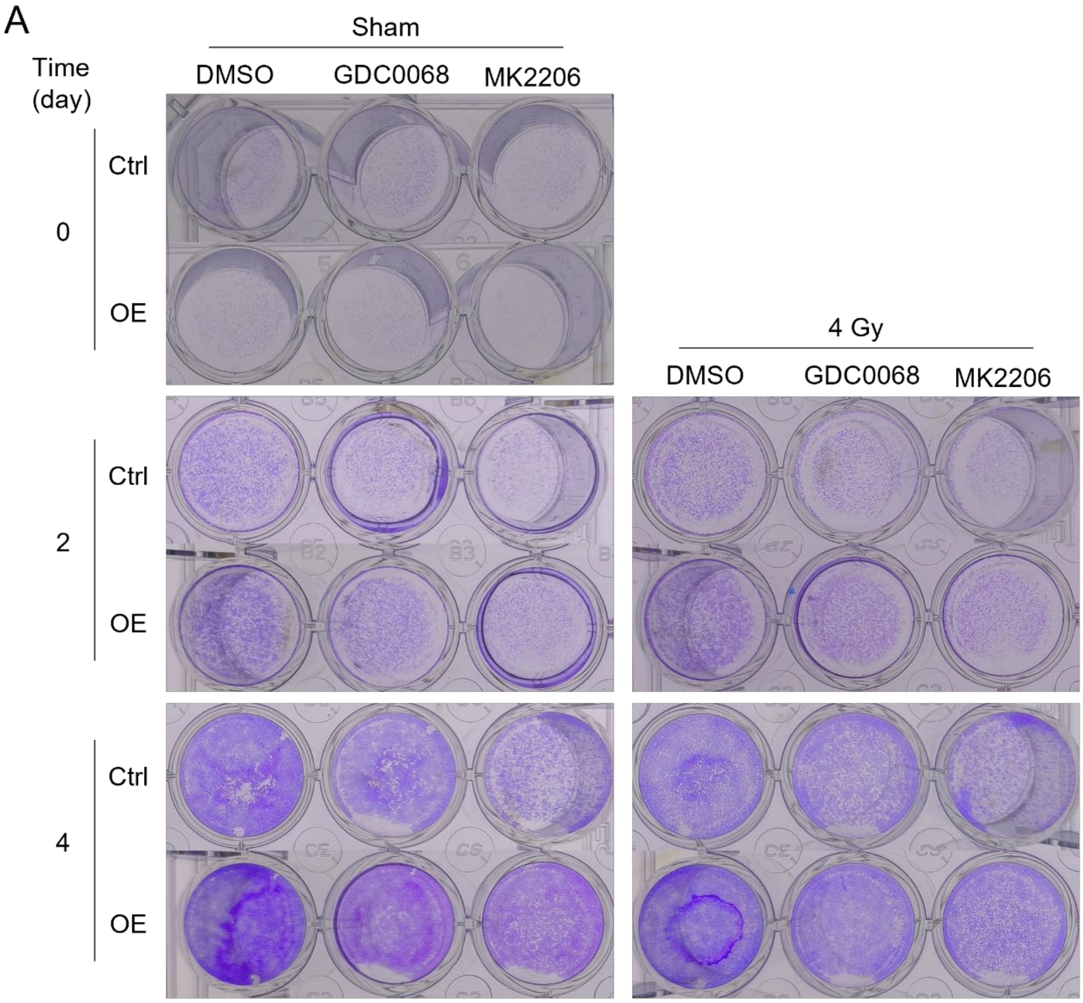

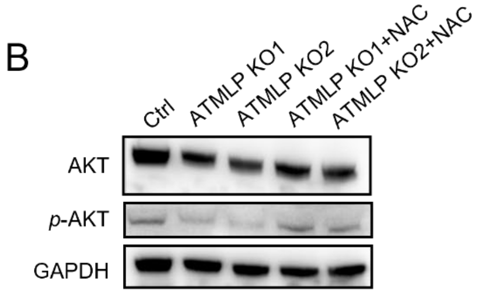

S5. (A) 5×10^4 A549 cells were inoculated into 6-well plates, and after the cells were stabilized, they were treated with AKT inhibitors and irradiated with 4 Gy X-rays after 24 hours. The cells were fixed and stained with crystal violet at 2 and 4 days after irradiation, respectively. The experiments were all repeated three times. (B) Western Blot assay to detect AKT, phosphorylated AKT (p-AKT) protein expression. GAPDH as an internal reference. NAC (N-acetylcysteine) was used at a working concentration of 5 mM to reduce intracellular ROS levels.

SUP 6.

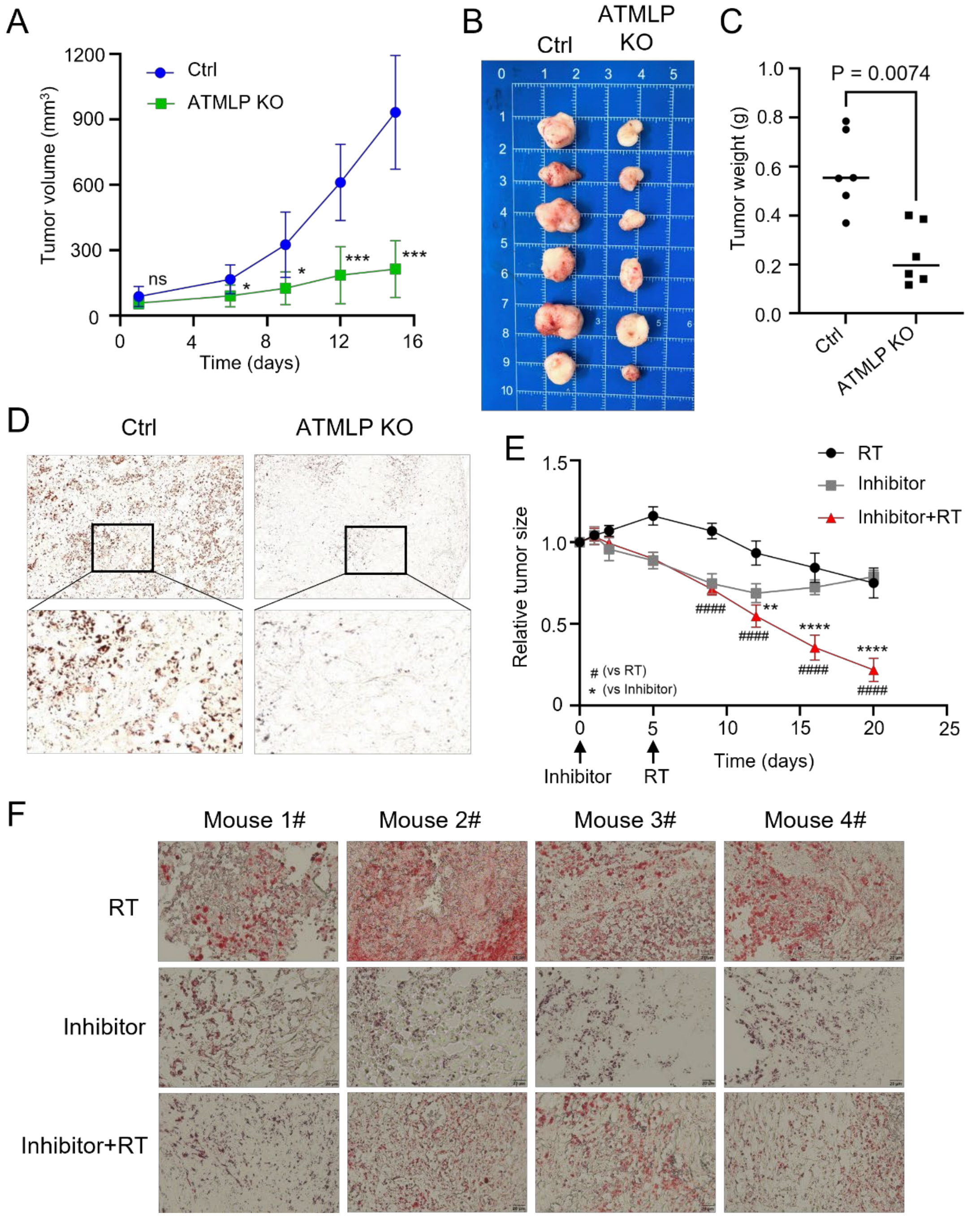

S6. Inhibition of ATMLP and AKT Expression Increases the Radiosensitivity of NSCLC In Vivo. (A) A xenograft model was established by subcutaneous injection of 1×10^6 H1299 cells into the buttocks of 8-week-old nude mice (n=6). Tumor size was observed and recorded. The horizontal and vertical markers are the time after cell inoculation, and the vertical coordinate is the tumor volume. (B) The mice were dissected at day 15, and the tumors were peeled out and photographed. (C) Mice were dissected at day 15, and the tumors were peeled out and weighed. (D) The xenografts were fixed and frozen sectioned, and the lipid droplet content was assessed by oil red staining. The figure below shows a local magnification. (E) Changes in xenograft volume in AKT inhibitor, radiation therapy and combined treatment mice. The experimental protocol was as described in Fig 7D. (F) On the 20th day of graft treatment, mice were euthanized, tumors were excised, and frozen

sections were stained with oil red. All animal experiments were approved by the Institutional Review Board (IRB) and the Animal Care and Use Committee (ACUC) of Soochow University, and were conducted in the SPF Animal Laboratory of Soochow University, in accordance with the ethical guidelines for animal welfare.
